# Quadriceps force and anterior tibial force occur obviously later than vertical ground reaction force: a simulation study

**DOI:** 10.1186/s12891-017-1832-6

**Published:** 2017-11-18

**Authors:** Ryo Ueno, Tomoya Ishida, Masanori Yamanaka, Shohei Taniguchi, Ryohei Ikuta, Mina Samukawa, Hiroshi Saito, Harukazu Tohyama

**Affiliations:** 1 Faculty of Health Sciences, Hokkaido University, North 12, West 5, Kitaku, Sapporo, 060-0812 Japan; 2 Hachioji Sports Orthopaedic Clinic, Hachioji-Nakacho-Bldg3, 5-1, Nakacho, Hachioji, Tokyo 192-0085 Japan

**Keywords:** Anterior cruciate ligament, Biomechanics, Musculoskeletal model, Quadriceps

## Abstract

**Background:**

Although it is well known that quadriceps force generates anterior tibial force, it has been unclear whether quadriceps force causes great anterior tibial force during the early phase of a landing task. The purpose of the present study was to examine whether the quadriceps force induced great anterior tibial force during the early phase of a landing task.

**Methods:**

Fourteen young, healthy, female subjects performed a single-leg landing task. Muscle force and anterior tibial force were estimated from motion capture data and synchronized force data from the force plate. One-way repeated measures analysis of variance and the post hoc Bonferroni test were conducted to compare the peak time of the vertical ground reaction force, quadriceps force and anterior tibial force during the single-leg landing. In addition, we examined the contribution of vertical and posterior ground reaction force, knee flexion angle and moment to peak quadriceps force using multiple linear regression.

**Results:**

The peak times of the estimated quadriceps force (96.0 ± 23.0 ms) and anterior tibial force (111.9 ± 18.9 ms) were significantly later than that of the vertical ground reaction force (63.5 ± 6.8 ms) during the single-leg landing. The peak quadriceps force was positively correlated with the peak anterior tibial force (*R* = 0.953, *P* < 0.001). Multiple linear regression analysis showed that the peak knee flexion moment contributed significantly to the peak quadriceps force (*R* ^2^ = 0.778, *P* < 0.001).

**Conclusion:**

The peak times of the quadriceps force and the anterior tibial force were obviously later than that of the vertical ground reaction force for the female athletes during successful single-leg landings. Studies have reported that the peak time of the vertical ground reaction force was close to the time of anterior cruciate ligament (ACL) disruption in ACL injury cases. It is possible that early contraction of the quadriceps during landing might induce ACL disruption as a result of excessive anterior tibial force in unanticipated situations in ACL injury cases.

## Background

Anterior cruciate ligament (ACL) injury is the most serious, common, and costly injury in young athletes [1]. It is estimated that 80,000 to more than 250,000 ACL injuries occur each year in the U.S. [2]. ACL reconstruction is a common treatment, and approximately 100,000 reconstructions are performed annually in the U.S. [2]. The direct cost of an ACL reconstruction in the U.S. was almost $12,000–17,000, making ACL reconstruction responsible for over $1 billion of the national health care costs [3, 4]. Furthermore, 67% of patients cannot return to competitive sports by 12 months post surgery [5]. Patients also require a long postoperative rehabilitation period after their ACL reconstruction. Therefore, effective ACL prevention programs are urgently needed. To resolve this problem, it is necessary to understand the ACL injury mechanism in more detail.

Seventy percent of ACL injuries result from a non-contact situation, such as jump landings and cutting tasks [6]. Previous in vitro studies have shown the effect of the external load and muscle force on the ACL force and strain [7–15]. Quadriceps force is commonly known to induce anterior tibial drawer and increase the ACL load [8, 9]. DeMorat et al. [10] reported that 4500 N of isolated quadriceps force produced significant anterior tibial translation with ACL rupture. It has been considered that high quadriceps force is a mechanism of ACL injury [10, 16].

Under anterior tibial force, the tension of the ACL is higher in the low angle of knee flexion [8, 17]. Therefore, an immediate increase in anterior tibial force after foot contact would present a greater risk of ACL injury. Kiapour et al. [14] suggested that peak ACL strain occurred at approximately 45 ms with maximum anterior tibial translation during a simulated single-leg drop landing using a cadaveric biomechanical testing apparatus. Although it is well known that quadriceps force generates anterior tibial force in the low angle of knee flexion [18], it is unclear whether quadriceps force causes great anterior tibial force during the early phase of a landing task [19]. Quadriceps force generates the internal knee extension moment to resist the external knee flexion moment during a landing task. The knee flexion moment is likely greater during the late phase because of the increase in knee flexion angle during landing [20]. Therefore, it is possible that the quadriceps force is greater during the late phase of landing. However, in vivo case studies and in vitro biomechanical studies have suggested greater vertical ground reaction force also causes a greater ACL load [11, 15, 21]. In their video research, Koga et al. [22] indicated that ACL injury occurred with a peak ground reaction force based on the estimated center of mass accelerations after initial foot contact with the ground. However, while the video research provided an estimation of the knee kinematics of ACL injury, it has not been shown how muscle force induces ACL injury [22–26].

A musculoskeletal modeling approach has been used to examine the effect of muscle force on ACL loading during landing tasks [27–31]. This approach is useful for estimating the muscle force during in vivo dynamic motions, which was not revealed in the video research or in vitro biomechanical testing. Focusing on the muscle force provides new insight into how the muscle force around the knee contributes to knee joint force, such as anterior tibial force, during landing tasks. Previous studies have been validated by comparisons with surface electromyography (EMG) and muscle activation (MA) estimated using the musculoskeletal model [27–30]. Therefore, this numerical model may suggest new insights regarding whether quadriceps contraction generates great anterior tibial force during the early phase of a landing task.

The purposes of the present study were (i) to compare the peak time of the vertical ground reaction force, quadriceps force and anterior tibial force during a landing task; (ii) to examine the relationship between peak quadriceps force and anterior tibial force; and (iii) to examine the contribution of experimental variables to the peak quadriceps force during the landing. The hypothesis of this study was that (i) the peak times of the quadriceps force and anterior tibial force were later than that of the vertical ground reaction force, (ii) the quadriceps force was correlated with the anterior tibial force and (iii) the knee flexion moment and knee flexion angle significantly contributed to quadriceps force.

## Methods

### Subjects and experimental task

**Fig. 1:**
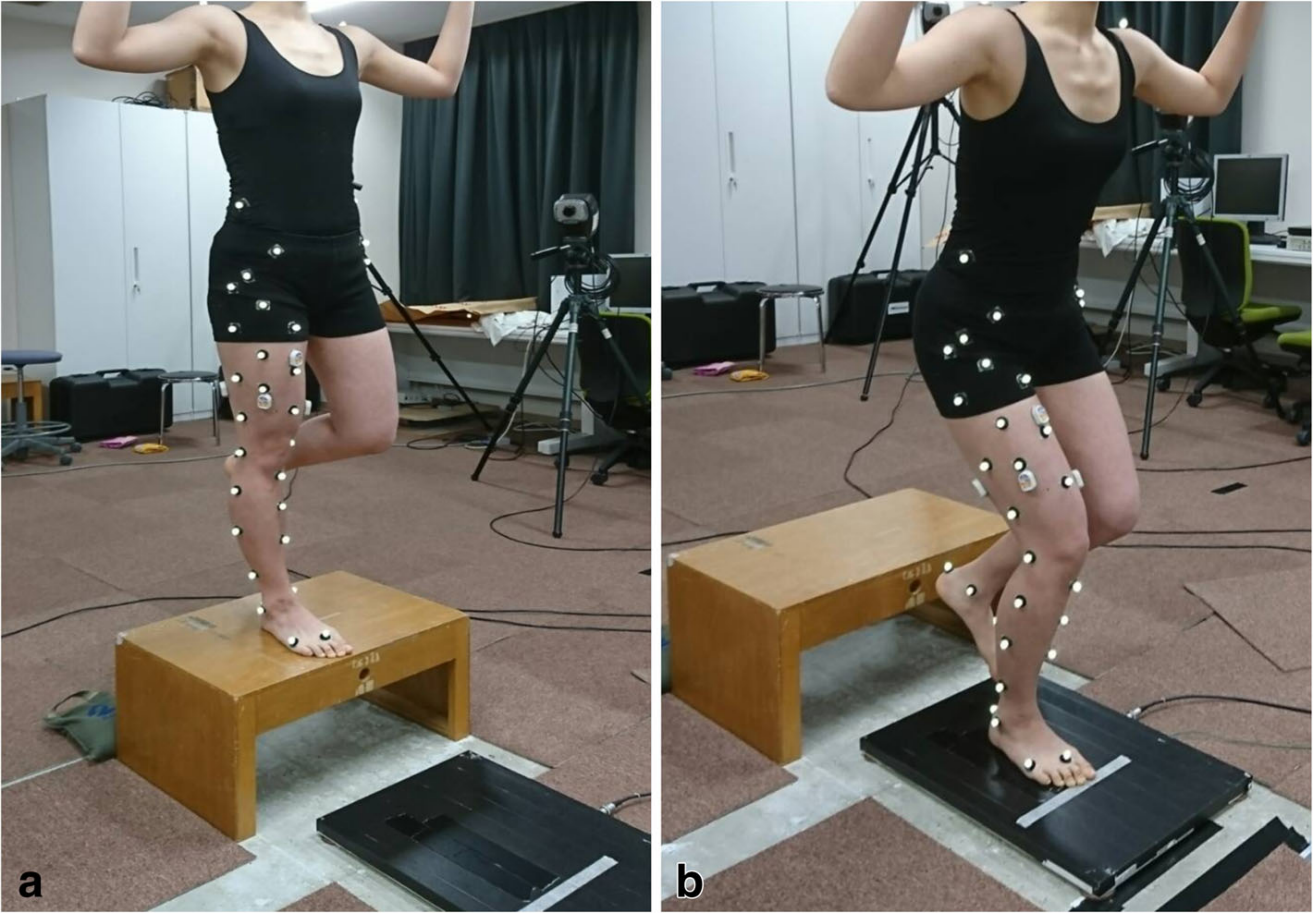
Motion capture during the single-leg landing trial. The subjects stood on their right leg on a 30-cm-high box (**a**) and dropped off on to a force plate, landing on their right leg (**b**)

Fourteen young, healthy, female subjects (age 21.5 ± 0.8 years, height 162.1 ± 5.9 cm, mass 53.2 ± 6.6 kg) participated in the present study. Each participant performed three trials of single-leg landing after sufficient instruction. The participants were instructed to stand on a 30-cm-high box with their preferred leg and drop off the box onto a force plate (Type 9286, Kistler AG, Winterthur, Switzerland), landing on the same leg (right leg for all, Fig. 1). Institutional review board approval and informed consent were obtained before the present study was conducted. The participants were accepted into the study if they had no history of lower extremity injury requiring surgical repair and had not suffered a knee injury within the previous 6 months. No subjects had any history of intrinsic bone disorders, metabolic disease, hormonal abnormalities or myogenic abnormalities, nor were they taking any medications. In addition, no subjects had excessively high or low muscle mass, and they did not demonstrate evident laxity or stiffness based on clinical orthopedic testing (e.g., Lachman test, pivot shift test and valgus stress test).

### Study procedure overview

**Fig. 2:**
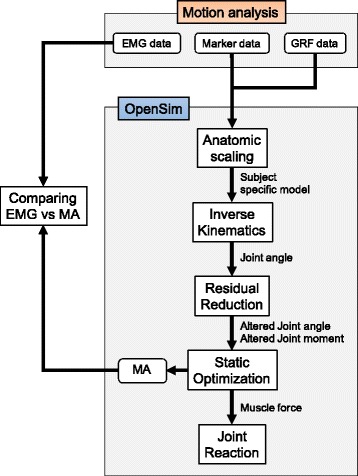
Flow chart for data processing, from motion capture to OpenSim analyses. GRF: ground reaction force, EMG: electromyography, MA: muscle activation

The overall data collecting and processing procedure is shown in Fig. 2. This study involved two distinct parts: the first comprised motion analysis trials in which the 14 female subjects performed the single-leg landing to obtain the marker trajectories, ground reaction force and EMG data. The second part was the numerical simulation of each trial with OpenSim 3.2, an open-source software [32], using kinematic and kinetic data from the motion analysis. To obtain the joint kinematics, muscle force and joint reaction force, a sequence of processes including anatomic scaling, inverse kinematics (IK), residual reduction algorism (RRA), static optimization (SO) and joint reaction analysis was performed. Finally, to validate the simulation, the wave patterns of the MA of quadriceps estimated with OpenSim were compared to those of the EMG from the motion analysis.

### Data collection

**Fig. 3:**
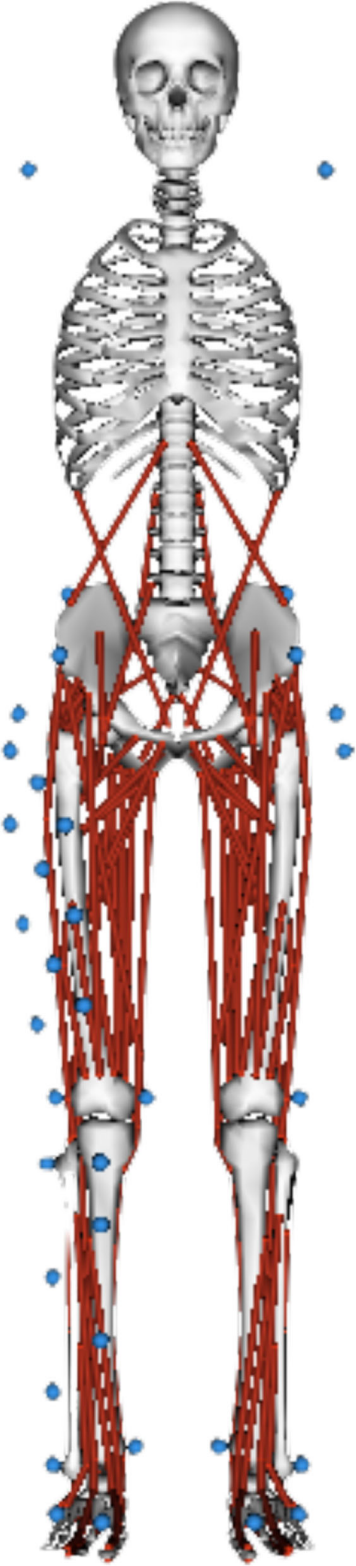
Generic musculoskeletal model and marker placements

Thirty-nine reflective markers were placed at strategic anatomical locations to obtain the knee kinematics (Fig. 3) [33]. The markers’ trajectories were collected using the EvaRT 4.4 motion capture system (Motion Analysis Corporation, Santa Rosa, CA, USA) and six digital cameras (Hawk cameras; Motion Analysis Corporation) at 200 Hz. Ground reaction forces were synchronously recorded at 1000 Hz using the force plate. Both kinematic and ground reaction force data ware low-pass filtered using a zero-lag fourth order Butterworth filter at 12 Hz.

The EMG data were measured using a wireless surface EMG system (WEB-1000; Nihon Kohden Corporation, Tokyo, Japan) with a sampling rate of 1000 Hz. The electrodes were placed on the rectus femoris, vastus medialis, and vastus lateralis of each participant’s right leg. All electrode positionings and related procedures were performed according to the SENIAM recommendations [34]. The raw experimental EMG data were band-pass filtered using a zero-lag fourth order Butterworth filter at cutoff frequency of 20 to 500 Hz and then full-wave rectified and low-pass filtered using a zero-lag fourth order Butterworth filter at a cutoff frequency of 12 Hz. Finally, peak EMG magnitudes for each trial were used to normalize the smoothed EMG data.

### Musculoskeletal models

Subject-specific musculoskeletal models were created by scaling the generic model, gait 2392, in OpenSim (Fig. 3). In the scaling process, the size, weight and inertial property of the subject-specific model were adjusted to those of the participants. The participants’ anthropometric measurements based on the marker positions and body weight measured during their static trial were used to scale the generic model. The generic model, gait 2392, had 23 degrees of freedom and 92 muscle-tendon actuators without any ligaments or upper extremity segments. According to previous studies [27, 28, 31], the maximum isometric forces for all muscles were scaled to twice those used by Delp et al. [35] to enable the muscle to resist the large external knee flexion moment during a single-leg landing. The strength of isometric muscle force of the subject-specific model was not scaled to that of each subject in the scaling process. After the model scaling, sequential IK, RRA, SO and joint reaction analyses were conducted.

The IK tool defined the joint kinematics using marker trajectories obtained from the motion capture system during the single-leg landing task. There were dynamical inconsistencies between the experimental joint kinematics from the IK and ground reaction force, an effect of modeling and marker data processing. RRA was conducted to alter the joint kinematics and the torso mass center of the subject-specific model to increase its consistency with the ground reaction force. Using the joint kinematics from RRA and ground reaction force data, the SO tool estimated the muscle force and activation. SO resolves the net joint moment into individual muscle forces by minimizing the sum of the squared MA at each time step. The step-driven Hill-type muscle model considers the force-length-velocity properties. Quadriceps force was defined as the combination of the rectus femoris and vasti muscle forces. Finally, a joint reaction analysis computed the internal joint force results using muscle force and external loads. The anterior component of the knee joint force in the tibial frame was defined as the anterior tibial force. All variables, EMG and MA were averaged using data from three successful trials.

### Data reduction

Foot strike was defined as the moment at which vertical ground reaction force reached just above 10 N. The landing phase was defined as the period from foot strike to peak knee flexion. The peak values for the vertical and posterior ground reaction force, altered knee flexion angle and knee flexion moment from the RRA, quadriceps force and anterior tibial force during the landing phase were used for the following statistical analysis.

### Statistical analysis

The sample size for one-way repeated measures of analysis of variance (ANOVA) was calculated with a combined effect size *f* of 0.25 (medium), an α-level of 0.05 and a power of 0.8 in a priori power calculation. The calculated sample size was 12, and 14 subjects were recruited for this study. One-way repeated measures ANOVA and a post hoc Bonferroni test were conducted to compare the peak time of the vertical ground reaction force, anterior tibial force and quadriceps force during the single-leg landing.

Pearson’s correlation coefficients were calculated to reveal the relationships of the peak values of vertical and posterior ground reaction force, knee flexion angle, knee flexion moment and anterior tibial force with the peak quadriceps force. A stepwise multiple linear regression analysis was performed to predict the peak quadriceps force using vertical and posterior ground reaction force, knee flexion angle and knee flexion moment as independent variables. Statistical significance was set at *P* < 0.05 for all analyses using the IBM SPSS Statistics 19 software program (IBM, Chicago, IL, USA).

## Results

**Fig. 4:**
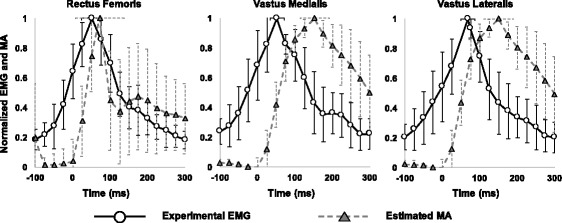
Comparisons of mean ± 1 standard deviation for experimental electromyography (EMG: circle and solid black line) and estimated muscle activation (MA: triangle and dashed gray line) during single-leg landing for all subjects. Both EMG and MA data were normalized by their peak values during landing. The horizontal error bar at the peak of the plot represents ±1 standard deviation for the peak time of the EMG and MA

The computationally estimated MA of the quadriceps showed fairly good consistency with collected experimental EMG findings (Fig. 4). Some delays of MA compared with EMG results were consistent with the electromechanical delay observed between EMG measurements and force production [36].

**Fig. 5:**
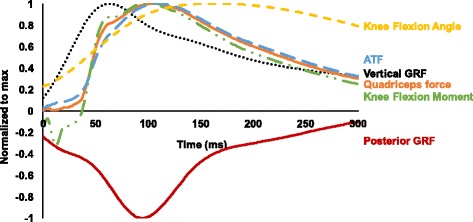
Time-history graph of the vertical and posterior ground reaction force (GRF), knee flexion angle and moment, quadriceps force and anterior tibial force

**Fig. 6:**
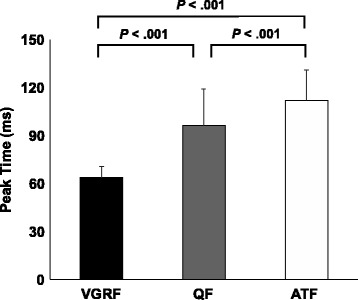
Comparison of the peak time of the vertical ground reaction force (VGRF), quadriceps force (QF) and anterior tibial force (ATF)

The time-history graph of normalized vertical and posterior ground reaction force, quadriceps force, anterior tibial force, knee flexion angle and knee flexion moment for all subjects is shown in Fig. 5, and the peak values of the variables are shown in Table 1. The mean values of the peak time for each force were 63.5 (6.8) ms, 96.0 (23.0) ms and 111.9 (18.9) ms after the initial foot contact for vertical ground reaction force, quadriceps force and anterior tibial force, respectively. The peak times of the estimated quadriceps force and anterior tibial force were significantly later than that of vertical ground reaction force during the single-leg landing (*P* < 0.001). The peak time of the anterior tibial force was also later than the peak quadriceps force (*P* < 0.001) (Fig. 6).

**Table 1:** Mean (SD) values of the kinetic and kinematic variables

Variables	Mean (SD)
Vertical ground reaction force (N)	1619 (148)
Posterior ground reaction force (N)	−218 (33)
Quadriceps force (N)	3741 (774)
Anterior tibial force (N)	3613 (836)
Knee flexion moment (Nm)	143 (29)
Knee flexion angle (deg)	55.6 (5.2)

**Fig. 7:**
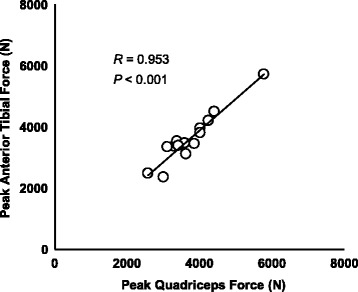
Correlation between the peak quadriceps force and anterior tibial force for all subjects

The Pearson’s correlation coefficient and the associated *P*-value for experimental variables and peak quadriceps force are given in Table 2. Peak quadriceps force was positively correlated with the peak anterior tibial force (*R* = 0.953, *P* < 0.001; Fig. 7).

**Table 2:** Correlations between kinetic and kinematic variables and peak quadriceps force

	Peak quadriceps force	
Variables	*R* ^a^	*P* value
Vertical ground reaction force (N)	0.519	0.057
Posterior ground reaction force (N)	−0.551	0.041
Knee flexion moment (Nm)	0.882	< 0.001
Knee flexion angle (deg)	0.505	0.065

^a^Pearson’s correlation coefficients

The multiple regression model showed that the peak knee flexion moment contributed significantly to maximum quadriceps force (*P* < 0.001, *R* ^2^ = 0.778). The estimated regression model for peak quadriceps force was

$$ \mathrm{Peak}\  \mathrm{quadriceps}\  \mathrm{force}=360.8+23.69\ \left(\mathrm{peak}\  \mathrm{knee}\  \mathrm{flexion}\  \mathrm{moment}\right) $$

The *P* value of the variables included in the regression model was *P* = 0.510. For the intercept and peak knee flexion moment, we calculated *P* < 0.001.

## Discussion

The purposes of the present study were to determine whether the peak time of the quadriceps force and anterior tibial force occur immediately after landing, to examine the relationship between quadriceps force and anterior tibial force and to examine the contribution of experimental variables to the peak quadriceps force during the single-leg landing task. The results supported our hypothesis that peak quadriceps force occurred at a later phase than peak vertical ground reaction force during the single-leg landing task. In addition, the results indicated that the quadriceps force generates greater anterior tibial force in the late phase during single-leg landing. The quadriceps force was predicted by an increase in the knee flexion moment.

EMG data were collected to compare the experimental EMG and the MA estimated using the musculoskeletal modeling approach. Fairly good consistencies between the EMG and MA were found. The comparison of the EMG and MA suggested that the simulation would be complete. In addition, wave patterns of the knee flexion moment, which contribute significantly to quadriceps force, and the other kinetic, kinematic data (knee flexion angle, vertical and posterior ground reaction force) were consistent with those previously reported for single-leg landing [20, 27, 28, 31, 37]. Therefore, the predicted muscle force could be reasonably used to examine the peak time of the quadriceps force for landing.

The present study showed that the peak times of the estimated quadriceps force and anterior tibial force were significantly later than that of vertical ground reaction force during successful single-leg landings for the young female athletes. The peak time of the anterior tibial force was significantly later but close to the peak time of the quadriceps force. The peak time of the quadriceps force and anterior tibial force were also obviously later than the time of maximum anterior tibial translation during in vitro simulated single-leg landing, as Kiapour et al. [14] previously reported, while the peak time of the vertical ground reaction force was close to the time of ACL disruption in ACL injury cases [22]. Therefore, it is possible that earlier and greater activation of the quadriceps during landing might induce ACL disruption via excessive anterior tibial force under unanticipated circumstances in ACL injury cases. Further studies should be conducted to clarify the time-sequence of quadriceps muscle forces and the ACL disruption during landing in ACL injury cases.

The regression analysis showed that the peak knee flexion moment contributed significantly to the peak quadriceps force. Although peak knee flexion angle was included in the regression model with stepwise selection, it was not significant. Thus, the peak flexion angle was excluded from the regression model. The wave pattern of the knee flexion moment was close to that of quadriceps force (Fig. 5). One key finding of this study is that quadriceps force is necessary to resist the greater external knee flexion moment during the late phase of landing. Shimokochi et al. [20] researched the effect of the several sagittal plane body postures - the trunk leaning forward with landing on the toes, upright trunk position with landing on the heel and a participant-selected position – on the knee extensor moment during single-leg landing. No significant differences in peak internal knee extensor moment were detected among the postures. In addition, there were likely minimal differences in the time to peak knee extensor moment. The previous results and the present findings suggest that the peak quadriceps force will occur during the late phase even if trunk and ankle postures are altered during single-leg landing. Therefore, quadriceps force may not be greater at the time of the ACL rupture during an anticipated landing.

There were several limitations of this study. First, the load of the ACL and the other ligaments were not estimated in the manner reported in previous studies [27–30, 38]. Estimating the ACL load is useful for evaluating the injury risk directly, but we focused on the peak time of the quadriceps force, and that depended on the knee flexion moment. The purposes of the present study were well addressed without evaluating the ACL load. Second, a posterior tibial slope was not included in the generic model. The posterior tibial slope induces an anterior tibial force, altering the vector of the compressive load anteriorly on the tibial plateau. The effect of the vertical ground reaction force on the anterior tibial force would have been greater if the posterior tibial slope had been modeled, as Shimokochi et al. suggested [20]. Third, the same strength of muscles was used for all subject-specific models without adjusting to that of each individual participant. Finally, it is unclear how the quadriceps is activated in ACL injury situations when the peak muscle force occurred during the late phase of successful single-leg landings. The knee flexion moment or muscle activation and the other kinetics have never been reported for ACL injury situations, with the exception of the estimated vertical ground reaction force [22]. In future studies, researching the knee flexion moment in cases of ACL injury may be useful to predict the quadriceps force contribution to ACL injury.

## Conclusions

We examined whether quadriceps force generates great anterior tibial force during the early phase of single-leg landing. The peak time of the quadriceps force during the single-leg landing is obviously later than the time at which ACL injury occurred in previous reports. In addition, the knee flexion moment contributed significantly to the quadriceps force in a linear regression model.

## Abbreviations

ACL: Anterior cruciate ligament
EMG: Electromyography
IK: Inverse kinematics
MA: Muscle activation
RRA: Residual reduction algorism
SO: Static optimization
